# Efficacy and Prognostic Factors for PARP Inhibitors in Patients With Ovarian Cancer

**DOI:** 10.3389/fonc.2020.00958

**Published:** 2020-06-16

**Authors:** Xuan-zhang Huang, Han Jia, Qiong Xiao, Run-zhou Li, Xing-shuang Wang, Hai-yan Yin, Xin Zhou

**Affiliations:** ^1^Key Laboratory of Precision Diagnosis and Treatment of Gastrointestinal Tumors, Department of Surgical Oncology and General Surgery, Ministry of Education, The First Affiliated Hospital of China Medical University, Shenyang, China; ^2^Department of Obstetrics and Gynecology, Shengjing Hospital of China Medical University, Shenyang, China

**Keywords:** PARP inhibitors, prognostic factor, BRCA mutation, homologous recombination deficiency, ovarian cancer

## Abstract

**Background:** The prognostic factors for efficacy of poly(ADP-ribose) polymerase (PARP) inhibitors in ovarian cancer remain unknown. The purpose of this study is to evaluate the efficacy of PARP inhibitors and to explore their prognostic factors in ovarian cancer.

**Methods:** PubMed, Embase, and conference databases were searched for relevant prospective clinical trials. The primary outcomes included overall survival (OS), progression-free survival (PFS), and their prognostic factors. Secondary outcomes included PFS2, time to first subsequent therapy (TFST), time to second subsequent therapy (TSST), chemotherapy-free interval (CFI), and their prognostic factors. Hazard ratio (HR) with a 95% confidence interval (CI) was used as an effect measure.

**Results:** PARP inhibitors significantly prolonged PFS in patients with ovarian cancer regardless of their BRCA and HRD status (HR = 0.44, 95% CI = 0.36–0.55). BRCA mutation, HRD-positive status, and sensitivity to platinum represented effective prognostic factors for PFS (P_interaction_ < 0.01 and within-trial interaction HR < 1). Other clinicopathological factors did not predict the benefit of PFS (P_interaction_ > 0.10). Moreover, PARP inhibitors significantly increased PFS2, TFST, TSST, and CFI, with significant BRCA-related differences. However, HRD-related differences could not be evaluated due to the lack of eligible studies. Furthermore, PARP inhibitors did not translate into prolonged OS, although there was a benefit associated with OS (HR = 0.84, 95% CI = 0.70–1.02). PARP inhibitors used as maintenance therapy after first or subsequent line therapy improved OS (HR = 0.77, 95% CI = 0.63–0.93).

**Conclusions:** PARP inhibitors can significantly prolong PFS, PFS2, TFST, TSST, and CFI in ovarian cancer patients. BRCA mutation, HRD-positive status, and sensitivity to platinum are effective prognostic factors for the efficacy of PARP inhibitors. However, despite the PFS improvement, this does not translate into prolonged OS for patients.

## Introduction

Ovarian cancer is a leading cause of death from gynecological cancers among females worldwide (1). The use of platinum- and taxane-based drugs is a milestone in the treatment of ovarian cancer. Cytoreductive surgery and systemic platinum-taxane combination chemotherapy have become the standard treatment for ovarian cancer (2). Although most patients with ovarian cancer have good initial responses to the first-line platinum-taxane combination chemotherapy, this response is not sustained in the majority of patients who still ultimately experience disease relapse and progression (3–5). Platinum-sensitive and platinum-resistant patients are defined by relapse duration >6 and <6 months after first-line platinum-based chemotherapy, respectively, and chemosensitivity to platinum-based chemotherapy is a predictive factor for patient survival and also a significant determinant for subsequent treatment. Unfortunately, there is little progress in the field of first-line therapy. After subsequent treatments with chemotherapeutic drugs, relapsed patients will eventually develop resistance to chemotherapy (2, 3). Thus, long-term survival for ovarian cancer patients remains poor. With increasing research on genetic aspects of ovarian cancer, it is clinically important to introduce new drugs based on new targets and corresponding treatment approaches for ovarian cancer treatments.

Poly(ADP-ribose) polymerase (PARP) is mainly involved in the repair of single-stranded DNA breaks and is an important and most-studied DNA repair enzyme in ovarian cancer (6). Pre-clinical evidence has shown that PARP can also modulate the repair of double-stranded DNA breaks when cancer cells have a homologous recombination deficiency (HRD) without the capacity to repair double-stranded DNA breaks (7, 8). Therefore, PARP inhibitors can result in apoptosis and cell death in the HRD cancer cells via a process of synthetic lethality, because PARP inhibitors prevent the repair of single-stranded DNA breaks and promote the conversion of single-stranded breaks to cytotoxic double-stranded breaks by trapping PARP at the sites of single-stranded breaks (9–11). Many clinical trials have been conducted to evaluate the efficacy and safety of PARP inhibitors for the treatment of ovarian cancer. Study 19 first showed that olaparib used as a maintenance treatment significantly improved progression-free survival (PFS) among patients with platinum-sensitive, relapsed, high-grade serous ovarian cancer (hazard ratio [HR] = 0.35, 95% CI = 0.25–0.49), while patients with BRCA-mutants had a greater PFS benefit from olaparib (HR = 0.18, 95% CI = 0.10–0.31) compared to patients with BRCA-wild (HR = 0.54, 95% CI = 0.34–0.85) (12, 13). SOLO1 trial also showed that maintenance therapy with olaparib after first-line platinum-based chemotherapy provided a substantial PFS benefit among patients with newly diagnosed advanced ovarian cancer and a BRCA1/2 mutation (HR = 0.30, 95% CI = 0.23–0.41) (14). These results showed that PARP inhibitors can significantly improve PFS and have good safety profiles in patients with BRCA-mutated ovarian cancer (14–16). Thus, PARP inhibitors have become an attractive treatment option and changed the therapeutic landscape for patients with BRCA-mutated ovarian cancer. This treatment strategy with PARP inhibitors for ovarian cancer is guided by a genetic biomarker.

Unfortunately, most clinical studies have ignored the importance of prognostic factors for the efficacy of PARP inhibitors in ovarian cancer and have, therefore, not explored which clinicopathological tumor factors can act as prognostic factors for PARP inhibitors. Aside from BRCA mutations, there are few favorable prognostic factors that guide the use of PARP inhibitors in clinical practice. Most studies on PARP inhibitors in ovarian cancer have mainly focused on the patients with BRCA mutations. However, it is not enough that only the BRCA mutation status becomes a clinical genetic indication for the use of PARP inhibitors. This will prevent PARP inhibitors from being extended to a larger group of ovarian cancer patients, considering the fact that ~50% of serous ovarian cancers carry an HRD (17), while the BRCA mutation is only identified in ~22% of ovarian cancer patients (18). Therefore, several recent studies, such as PAOLA-1 and PRIMA, have begun to focus on the effect of HRD status on the efficacy of PARP inhibitors because HRD is more widespread in ovarian cancer than BRCA (19, 20). Furthermore, progression-free survival (PFS) is the primary outcome instead of overall survival (OS) in clinical trials on PARP inhibitors. Whether the improved PFS can translate into an OS benefit remains controversial.

Therefore, the purpose of this study was to evaluate the efficacy of PARP inhibitors in ovarian cancer, including PFS, OS, PFS2, time to first subsequent therapy (TFST), time to second subsequent therapy (TSST), and chemotherapy-free interval (CFI), and to explore whether clinicopathological factors can be used as prognostic factors for the efficacy of PAPR inhibitors.

## Materials and Methods

### Literature Search

A systematical literature search was performed for prospective clinical trials that evaluated PARP inhibitors' clinical efficacy and their prognostic factors for treatment and maintenance in patients with ovarian cancer by searching PubMed and Embase databases (up to February 2020). Society of Gynecologic Oncology, American Society of Clinical Oncology, European Society of Medical Oncology, and International Gynecologic Cancer Society Meeting Abstract were also searched for relevant trials. The search terms consisted of “olaparib,” “lynparza,” “rucaparib,” “rubraca,” “niraparib,” “zejula,” “talazoparib,” “talzenna,” “veliparib,” “iniparib,” “pamiparib,” “fluzoparib,” “poly ADP ribose polymerase inhibitor,” “PARP inhibitor,” “CEP8983,” “IMP4297,” “ovarian cancer,” “ovarian carcinoma,” “ovarian neoplasm,” “ovarian tumor,” and “ovarian malignancy.” In addition, reference lists for the relevant studies were manually searched for other potential studies.

### Eligibility Criteria and Data Extraction

Studies were included in the meta-analysis if they met all of the following eligibility criteria: (1) participants: patients were diagnosed with ovarian cancer and were ≥18 years old; (2) intervention: PARP inhibitors were administered alone or in combination with another chemotherapy, regardless of treatment or maintenance setting; (3) comparison: any treatment regimens that did not contain PARP inhibitors, including placebo and other chemotherapy treatments without PARP inhibitors. Studies that only explored the prognostic factors rather than PARP inhibitor efficacy could be set without the above comparison group; (4) outcome: the primary outcomes were PFS, OS, and their prognostic factors, while the secondary outcomes were PFS2, TFST, TSST, CFI, and their prognostic factors; (5) study design: only prospective clinical trials were eligible. To obtain more data, the most informative studies were included. Data that were only published in excluded duplicated studies were also extracted for the meta-analysis when there were several studies based on the same population cohort.

Data extraction from eligible studies was independently conducted by two authors (Xuan-zhang Huang and Han Jia). For each eligible study, the extracted data consisted of first author, publication year, trial name, trial phase, sample size, age, follow-up time, ovarian cancer type, BRCA and HRD status, tumor stage, PARP inhibitor type, therapy type, therapy line, treatment regimens, and outcome measures containing PFS, OS, PFS2, TFST, TSST, and CFI. Any issues with data extraction were resolved by discussion.

### Statistical Analysis

HR with a corresponding 95% confidence interval (CI) was used as an effect measure to assess the efficacy and prognostic factors for PARP inhibitors in ovarian cancer. The HR and 95% CI were calculated from available data using the method designed by Tierney, if the values were not provided directly (21). LogHRs were calculated using a logarithm transformation of the HRs for each eligible study and the relative standard errors (SElogHR) of logHRs were calculated using the following formula: SElogHR = [(logUCI – logLCI)/(2 × invnorm(0.975))]. Subsequently, logHRs and SElogHR were used to obtain the pooled HR. Many of these eligible studies had several different endpoints. Thus, meta-analysis was performed based on different endpoints, including PFS, OS, PFS2, TFST, TSST, and CFI. For one study that reported multiple separate results based on different subgroups, multiple HR values were combined into a pooled HR value for further meta-analysis (22). The overall analysis was performed by including all eligible studies for every certain endpoint. To reduce the effect of different populations on the results, subgroup analysis was performed on the basis of PARP inhibitor type, BRCA and HRD status, response to platinum-based chemotherapy (CR or PR), platinum-free interval (PFI), therapy type, treatment regimens, therapy line, surgery type, residual disease status after surgery, tumor stage, patient age, performance-status score, and race. A random-effects model was used to pool the HRs because heterogeneity was present among clinical studies and a random-effects model was able to obtain more conservative results compared to a fixed-effects model (23). Heterogeneity among the studies was assessed using the Cochran's Q test and *I*^2^ statistics (24). Publication bias was assessed using Begg's and Egger's tests (25, 26). To take into consideration the influence of a single study on the results and to evaluate the consistency and stability of results, sensitivity analyses were performed using the leave-one-out approach in which meta-analysis was performed by omitting each study in turn.

To explore which clinicopathological factors could predict PARP inhibitor efficacy, interaction between the factors and PARP inhibitor efficacy was evaluated using an interaction test (27). To test the reliability and accuracy of the interaction results, a within-trial interaction HR (ratio of HRs in two different subgroups stratified by factors) was used to validate the differences in PARP inhibitor efficacy between different subgroups and then to identify which prognostic factors were significant and which subgroups could benefit more from PARP inhibitors (28, 29).

A two-sided *p* < 0.05 was considered statistically significant. All statistical analyses were conducted using Stata software (Version 12.0, Stata Corporation, College Station, TX, USA).

## Results

### Study Selection and Associated Characteristics

**Figure 1:**
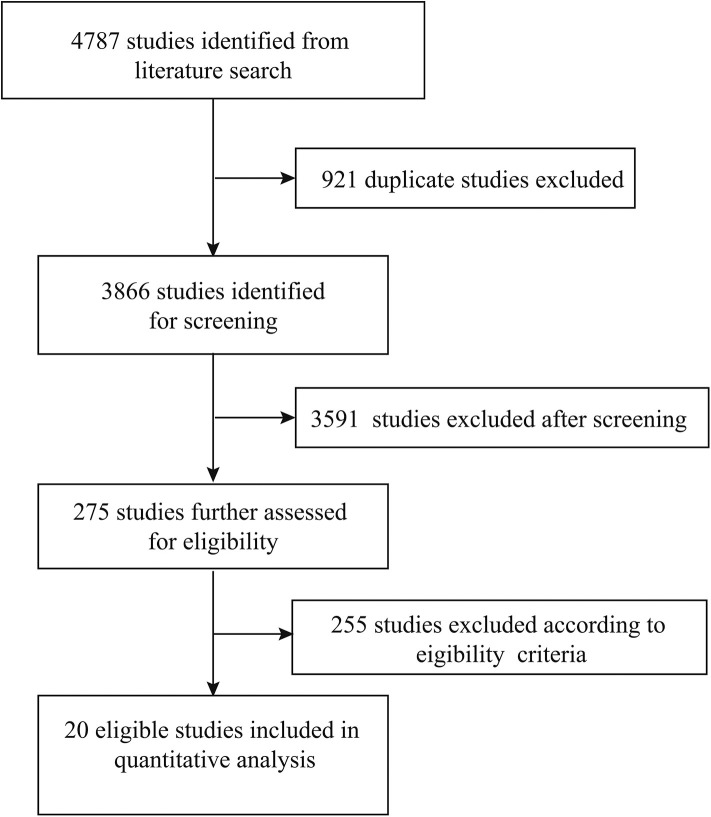
Literature search and study selection.

A total of 4,787 studies were originally identified from the literature search, of which 921 studies were excluded because of duplications. A total of 3,591 studies were then excluded after screening their titles and abstracts. The remaining 275 studies were further assessed based on a full-text review. A total of 255 studies were excluded based on eligibility criteria. Finally, 20 studies were included in the quantitative analysis (Figure 1) (14–16, 19, 20, 30–44).

A total of 20 studies published between 2011 and 2019 and containing 6,133 ovarian cancer patients were included in the present study. Of the eligible studies, 13 studies included patients with platinum-sensitive ovarian cancer, two studies included patients with platinum-resistant ovarian cancer, and five studies had a mix of platinum-sensitive and platinum-resistant, or unreported patients. Eleven studies evaluated olaparib, three studies evaluated rucaparib, three studies evaluated niraparib, and three studies evaluated veliparib in terms of the PARP inhibitor type. A total of seven studies used PARP inhibitors in a maintenance setting and 13 studies used PARP inhibitors in a treatment setting. With regard to the outcomes, PFS was available in 20 studies, OS in nine studies, PFS2 in six studies, TFST in eight studies, TSST in six studies, and CFI in two studies. The main baseline characteristics for the included studies are summarized in Table 1.

**Table 1:** The baseline characteristics of included studies.

**References**	**Trial phase**	**Cancer feature**	**Sample**	**Drug**	**Intervention**	**Control**
Coleman et al. (34)	Phase 3	Untreated, stage III or IV ovarian cancer	1,140	Veliparib	Carboplatin + paclitaxel + veliparib, followed by veliparib maintenance	Carboplatin + paclitaxel + placebo, followed by placebo maintenance
Penson et al. (31)	Phase 3	gBRCA-mutated, platinum-sensitive, relapsed ovarian cancer	266	Olaparib	Olaparib	Non-platinum chemotherapy(paclitaxel or topotecan or gemcitabine)
Gonzalez-Martin et al. (20)	Phase 3	Newly diagnosed, stage III or IV, platinum-sensitive ovarian cancer	733	Niraparib	Niraparib	Placebo
Ray-Coquard et al. (19)	Phase 3	Newly diagnosed, stage III or IV, platinum-sensitive ovarian cancer	806	Olaparib	Olaparib + bevacizumab	Placebo + bevacizumab
Mirza et al. (32)	Phase 2	Platinum-sensitive, recurrent ovarian cancer	97	Niraparib	Niraparib + bevacizumab	Niraparib
Colombo et al. (33)	Phase 2	Recurrent, platinum-resistant ovarian cancer	123	Olaparib	Olaparib + cediranib	Paclitaxel
Rivkin et al. (30)	Phase 1b	Advanced relapsed ovarian cancer	54	Olaparib	Olaparib + carboplatin + paclitaxel, followed by olaparib maintenance	Not applicable
Moore et al. (14)	Phase 3	Newly diagnosed, BRCA-mutated, stage III or IV, platinum-sensitive ovarian cancer	391	Olaparib	Olaparib	Placebo
Pujade-Lauraine et al. (15)	Phase 3	Platinum-sensitive, BRCA-mutated, relapsed ovarian cancer	295	Olaparib	Olaparib	Placebo
Coleman et al. (36)	Phase 3	Platinum-sensitive, high-grade, recurrent ovarian cancer	564	Rucaparib	Rucaparib	Placebo
Swisher et al. (16)	Phase 2	Relapsed, platinum-sensitive ovarian cancer	204	Rucaparib	Rucaparib	Not applicable
Oza et al. (35)	Phase 1/2	Relapsed, BRCA-mutated ovarian cancer	106	Rucaparib	Rucaparib	Not applicable
Ledermann et al. (39)	Phase 2	Platinum-sensitive, recurrent ovarian cancer	265	Olaparib	Olaparib	Placebo
Mirza et al. (38)	Phase 3	Platinum-sensitive, recurrent ovarian cancer	553	Niraparib	Niraparib	Placebo
Steffensen et al. (37)	Phase 1/2	Relapsed, gBRCA-mutated, platinum-resistant, or partially platinum-sensitive ovarian cancer	48	Veliparib	Veliparib	Not applicable
Oza et al. (40)	Phase 2	Platinum-sensitive, recurrent ovarian cancer	162	Olaparib	Olaparib + paclitaxel + carboplatin, followed by olaparib maintenance	Paclitaxel + carboplatin
Kummar et al. (41)	Phase 2	BRCA-mutated, recurrent ovarian cancer, or primary ovarian cancer regardless of BRCA mutation	75	Veliparib	Cyclophosphamide + veliparib	Cyclophosphamide
Liu et al. (42)	Phase 2	Platinum-sensitive, relapsed ovarian cancer	90	Olaparib	Olaparib + cediranib	Olaparib
Kaye et al. (43)	Phase 2	Recurrent, gBRCA-mutated ovarian cancer	97	Olaparib	Olaparib	Pegylated liposomal doxorubicin
Gelmon et al. (44)	Phase 2	Advanced ovarian cancer	64	Olaparib	Olaparib	Not applicable

### PFS for PARP Inhibitors

**Figure 2:**
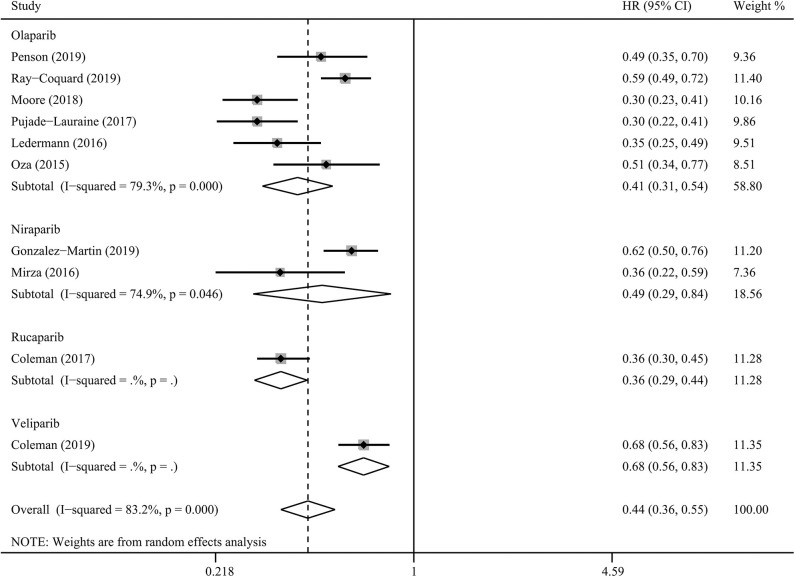
Progression-free survival benefit from PARP inhibitors in patients with ovarian cancer.

**Figure 3:**
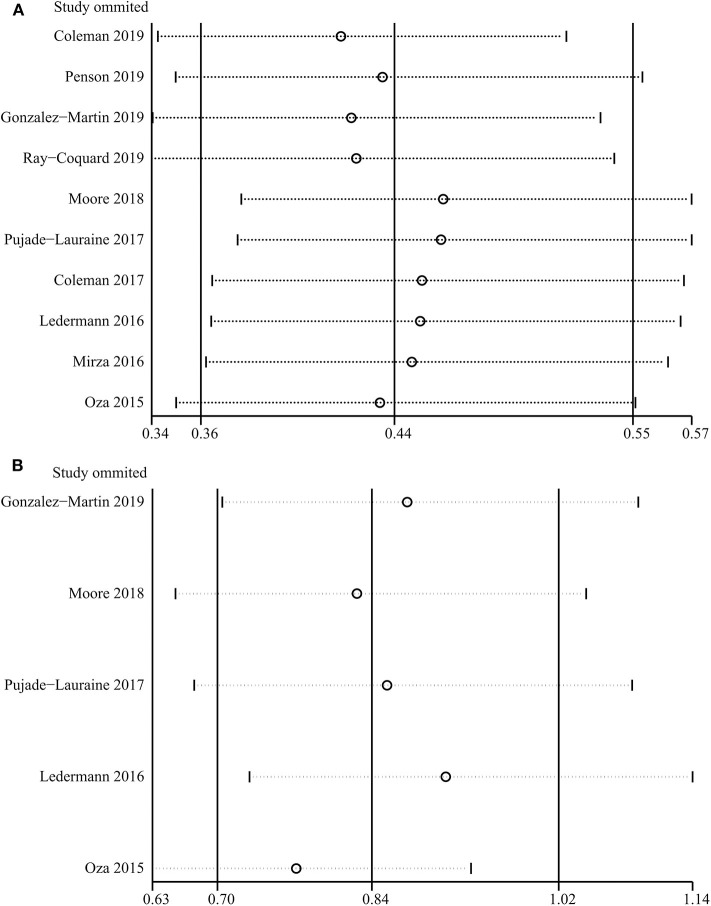
Sensitivity analysis of progression-free survival **(A)** and overall survival **(B)** based on leave-one-out approach.

PARP inhibitors significantly improved PFS in the whole group of patients with ovarian cancer regardless of BRCA and HRD status and therapy type (HR = 0.44, 95% CI = 0.36–0.55, Figure 2). Moreover, sensitivity analysis indicated that the result was not affected and dominated by any single trial, confirming the consistency and stability of the result (Figure 3). The subgroup analysis based on PARP inhibitors showed that olaparib, niraparib, veliparib, and rucaparib significantly prolonged PFS (Table 2). Subgroup analysis found a significant PFS benefit from PARP inhibitors in the BRCA mutation (HR = 0.31, 95% CI = 0.26–0.38), BRCA1 mutation, BRCA2 mutation, BRCA-wild (HR = 0.60, 95% CI = 0.48–0.76), HRD-positive (HR = 0.40, 95% CI = 0.32–0.50), and HRD-negative (HR = 0.74, 95% CI = 0.59–0.94) subgroups. PARP inhibitors used in combination with and without chemotherapy and in treatment and maintenance settings also had favorable PFS benefits. In addition, subgroup analysis based on response to platinum-based chemotherapy, PFI, therapy line, surgery type, residual disease status after surgery, tumor stage, patient age, performance-status score, and race obtained similar results, which indicated that PARP inhibitors were positively associated with a favorable PFS (Table 2).

**Table 2:** The results for the efficacy of PARP inhibitors in patients with ovarian cancer.

	**HR**	**95% CI**	**P**	**Publication biasl**
**PFS**				
Overall	0.44	0.36–0.55	<0.001	Begg's test = 0.721; Egger's test = 0.152l
**Drug type**				
Olaparib	0.41	0.31–0.54	<0.001	Begg's test = 0.452; Egger's test = 0.293l
Niraparib	0.49	0.29–0.84	0.009	Begg's test = 1.000; Egger's test = /l
**Therapy regimen**				
PARP inhibitor + chemo vs. chemo	0.62	0.54–0.71	<0.001	Begg's test = 1.000; Egger's test = 0.531l
PARP inhibitor vs. placebo	0.37	0.29–0.49	<0.001	Begg's test = 0.707; Egger's test = 0.355l
**Treat type**				
Treatment setting	0.58	0.46–0.73	<0.001	Begg's test = 1.000; Egger's test = 0.187l
Maintenance setting	0.40	0.31–0.52	<0.001	Begg's test = 1.000; Egger's test = 0.172l
≥2 line treatment	0.50	0.38–0.65	<0.001	Begg's test = 1.000; Egger's test = /l
Maintenance after 1 line therapy	0.49	0.33–0.72	<0.001	Begg's test = 1.000; Egger's test = 0.186l
Maintenance after ≥2 line therapy	0.34	0.30–0.40	<0.001	Begg's test = 1.000; Egger's test = 0.720l
Maintenance after ≥3 line therapy	0.33	0.26–0.42	<0.001	Begg's test = 1.000; Egger's test = /l
Maintenance after ≥4 line therapy	0.27	0.20–0.36	<0.001	Begg's test = 1.000; Egger's test = /l
**BRCA and HRD status**				
BRCA mutation	0.31	0.26–0.38	<0.001	Begg's test = 0.474; Egger's test = 0.466l
BRCA wild	0.60	0.48–0.76	<0.001	Begg's test = 1.000; Egger's test = 0.645l
BRCA1 mutation	0.38	0.30–0.48	<0.001	Begg's test = 0.296; Egger's test = 0.430l
BRCA2 mutation	0.24	0.10–0.59	0.002	Begg's test = 1.000; Egger's test = 0.453l
HRD positive	0.40	0.32–0.50	<0.001	Begg's test = 0.806; Egger's test = 0.964l
HRD negative	0.74	0.59–0.94	0.012	Begg's test = 0.027; Egger's test = 0.096l
BRCA wild + HRD positive	0.44	0.35–0.55	<0.001	Begg's test = 0.734; Egger's test = 0.916l
**Response to chemotherapy**				
Complete response	0.38	0.30–0.49	<0.001	Begg's test = 0.230; Egger's test = 0.127l
Partial response	0.41	0.30–0.57	<0.001	Begg's test = 0.368; Egger's test = 0.454l
**Age**				
<65 years	0.46	0.35–0.60	<0.001	Begg's test = 0.368; Egger's test = 0.297l
≥65 years	0.53	0.43–0.64	<0.001	Begg's test = 0.368; Egger's test = 0.324l
**Performance status**				
0	0.56	0.42–0.74	<0.001	Begg's test = 0.308; Egger's test = 0.161l
1	0.58	0.47–0.72	<0.001	Begg's test = 0.734; Egger's test = 0.325l
**Stage**				
III	0.53	0.39–0.71	<0.001	Begg's test = 0.089; Egger's test = 0.012l
IV	0.64	0.48–0.84	0.002	Begg's test = 1.000; Egger's test = 0.977l
**Residual disease after surgery**				
No macroscopic residual disease	0.45	0.33–0.60	<0.001	Begg's test = 0.308; Egger's test = 0.644l
Macroscopic residual disease	0.56	0.42–0.73	<0.001	Begg's test = 0.734; Egger's test = 0.638l
**Surgery timing**				
Primary surgery	0.50	0.32–0.77	0.002	Begg's test = 0.296; Egger's test = 0.144l
Interval surgery	0.56	0.40–0.77	<0.001	Begg's test = 0.296; Egger's test = 0.288l
**Platinum-free interval**				
>12 months	0.39	0.31–0.48	<0.001	Begg's test = 1.000; Egger's test = 0.918l
6–12 months	0.40	0.27–0.57	<0.001	Begg's test = 0.296; Egger's test = 0.480l
>6 months	0.38	0.32–0.44	<0.001	Begg's test = 0.260; Egger's test = 0.511l
**Previous bevacizumab use**				
YES	0.25	0.09–0.73	0.011	Begg's test = 1.000; Egger's test = /l
NO	0.35	0.28–0.44	/	/l
**Race**				
White	0.45	0.28–0.73	0.001	Begg's test = 1.000; Egger's test = 0.567l
Asian	0.48	0.32–0.71	<0.001	Begg's test = 0.296; Egger's test = 0.181l
Unknown	0.63	0.39–1.04	0.07	Begg's test = 0.734; Egger's test = 0.653l
**OS**				
Overall	0.84	0.70–1.02	0.075	Begg's test = 0.806; Egger's test = 0.615l
**Drug type**				
Olaparib	0.87	0.70–1.09	0.239	Begg's test = 1.000; Egger's test = 0.467l
**Therapy regimen**				
PARP inhibitor vs. placebo	0.77	0.63–0.93	0.008	Begg's test = 0.308; Egger's test = 0.493l
**Therapy type**				
Maintenance setting	0.77	0.63–0.93	0.008	Begg's test = 0.308; Egger's test = 0.493l
Maintenance after 1 line therapy	0.81	0.59–1.13	0.220	Begg's test = 1.000; Egger's test = /l
Maintenance after ≥2 line therapy	0.75	0.59–0.95	0.018	Begg's test = 1.000; Egger's test = /l
**BRCA and HRD status**				
BRCA mutation	0.78	0.61–1.01	0.058	Begg's test = 0.734; Egger's test = 0.351l
**PFS2**				
Overall	0.65	0.54–0.78	<0.001	Begg's test = 0.260; Egger's test = 0.177l
**Drug type**				
Olaparib	0.61	0.41–0.91	0.016	Begg's test = 1.000; Egger's test = 0.044l
Niraparib	0.70	0.54–0.90	0.006	Begg's test = 1.000; Egger's test = /l
**Therapy regimen**				
PARP inhibitor vs. placebo	0.61	0.52–0.71	<0.001	Begg's test = 0.806; Egger's test = 0.617l
**Therapy type**				
Maintenance setting	0.65	0.54–0.78	<0.001	Begg's test = 0.260; Egger's test = 0.177l
Maintenance after 1 line therapy	0.72	0.52–0.99	0.040	Begg's test = 0.296; Egger's test = 0.463l
Maintenance after ≥2 line therapy	0.60	0.50–0.70	<0.001	Begg's test = 0.296; Egger's test = 0.237l
**BRCA and HRD status**				
BRCA mutation	0.48	0.39–0.59	<0.001	Begg's test = 0.308; Egger's test = 0.515l
HRD positive	0.65	0.45–0.93	0.019	Begg's test = 1.000; Egger's test = /l
**TFST**				
Overall	0.44	0.35–0.55	<0.001	Begg's test = 0.536; Egger's test = 0.302l
**Drug type**				
Olaparib	0.41	0.30–0.57	<0.001	Begg's test = 0.806; Egger's test = 0.334l
Niraparib	0.57	0.38–0.84	0.005	Begg's test = 1.000; Egger's test = /l
**Therapy regimen**				
PARP vs. placebo	0.40	0.30–0.52	<0.001	Begg's test = 0.452; Egger's test = 0.385l
PARP+chemo vs. chemo	0.59	0.50–0.70	<0.001	Begg's test = 1.000; Egger's test = /l
**Therapy type**				
Maintenance setting	0.43	0.33–0.54	<0.001	Begg's test = 0.368; Egger's test = 0.186l
Maintenance after 1 line therapy	0.49	0.33–0.74	0.001	Begg's test = 1.000; Egger's test = 0.311l
Maintenance after ≥2 line therapy	0.37	0.30–0.46	<0.001	Begg's test = 0.734; Egger's test = 0.767l
**BRCA and HRD status**				
BRCA mutation	0.30	0.26–0.34	<0.001	Begg's test = 0.764; Egger's test = 0.100l
BRCA wild	0.45	0.34–0.60	<0.001	Begg's test = 1.000; Egger's test = /l
HRD positive	0.42	0.34–0.52	<0.001	Begg's test = 1.000; Egger's test = /l
**TSST**				
Overall	0.55	0.42–0.72	<0.001	Begg's test = 0.806; Egger's test = 0.672l
**Drug type**				
Olaparib	0.52	0.38–0.70	<0.001	Begg's test = 1.000; Egger's test = 0.841l
**Therapy regimen**				
PARP vs. placebo	0.51	0.39–0.66	<0.001	Begg's test = 0.089; Egger's test = 0.073l
**Therapy type**				
Maintenance setting	0.51	0.39–0.66	<0.001	Begg's test = 0.089; Egger's test = 0.073l
Maintenance after ≥2 line therapy	0.52	0.37–0.74	<0.001	Begg's test = 0.296; Egger's test = 0.229l
**BRCA and HRD status**				
BRCA mutation	0.42	0.35–0.49	<0.001	Begg's test = 1.000; Egger's test = 0.285l
BRCA wild	0.64	0.48–0.84	0.002	Begg's test = 1.000; Egger's test = /l
**CFI**				
Overall	0.43	0.35–0.53	<0.001	Begg's test = 1.000; Egger's test = /l
**BRCA and HRD status**				
BRCA mutation	0.28	0.21–0.37	<0.001	Begg's test = 1.000; Egger's test = /l

*HR, Hazard Ratio; CI, Confidence Interval; PFS, Progression-Free Survival; OS, Overall Survival; TFST, Time to First Subsequent Therapy; TSST, Time to Second Subsequent Therapy; CFI, Chemotherapy-Free Interval; HRD, Homologous Recombination Deficiency; chemo, chemotherapy. “/”, Not applicable due to limited number of studies*.

Interaction tests were performed because clinical practitioners have concerns about whether the efficacy of PARP inhibitors is affected by clinicopathological factors in patients with ovarian cancer. For the BRCA and HRD status, the results indicated that there was a significant difference in PFS improvement for PARP inhibitors between BRCA mutation and BRCA-wild (*P*_interaction_ < 0.001), between HRD-positive and HRD-negative (*P*_interaction_ < 0.001), between BRCA-wild with HRD-positive and BRCA mutation (*P*_interaction_ = 0.02), and between BRCA-wild with HRD-positive and HRD-negative (*P*_interaction_ = 0.002) groups (Table 3). No significant differences were present between BRCA mutation and HRD-positive (*P*_interaction_ = 0.088), and between BRCA1 and BRCA2 mutation (*P*_interaction_ = 0.327) subgroups. Moreover, no significant differences in PFS were observed in response to platinum-based chemotherapy (CR vs. PR), PFI, surgery type, residual disease status after surgery, tumor stage, patient age, performance-status score, and race (all *P*_interaction_ > 0.10, Table 3). Pooled within-trial interaction HRs confirmed the above interaction results, with more PFS benefits present in the BRCA mutation (HR = 0.55; 95% CI = 0.39–0.78) and HRD-positive (HR = 0.59; 95% CI = 0.43–0.81) groups. There were comparable PFS benefits in other clinicopathological factors (Table 3).

**Table 3:** Pooled within-trial interaction HRs for the progression-free survival of PARP inhibitors.

	***P*_interaction_**	**HR**	**95% CI**	***P*_HR_**	**Publication bias**
**BRCA and HRD status**					
BRCA mutation vs. BRCA wild	<0.001	0.55	0.39–0.78	0.001	Begg's test = 0.592; Egger's test = 0.515
HRD vs. non-HRD	<0.001	0.59	0.43–0.81	0.001	Begg's test = 0.707; Egger's test = 0.308
BRCA wild + HRD vs. BRCA mutation	0.020	1.41	1.03–1.93	0.033	Begg's test = 0.221; Egger's test = 0.016
BRCA wild + HRD vs. non-HRD	0.002	0.61	0.48–0.78	<0.001	Begg's test = 0.707; Egger's test = 0.648
BRCA1 vs. BRCA2	0.327	1.53	0.66–3.50	0.320	Begg's test = 1.000; Egger's test = 0.548
Platinum-sensitive vs. platinum-resistant	/	0.53	0.33–0.84	0.007	Begg's test = 1.000; Egger's test = 0.507
Response to chemotherapy: CR vs. PR	0.712	0.88	0.60–1.29	0.506	Begg's test = 1.000; Egger's test = 0.547
CA155: normal vs. abnormal	/	0.76	0.47–1.23	/	/
Age: <65 vs. ≥65 years	0.407	0.93	0.77–1.12	0.442	Begg's test = 0.230; Egger's test = 0.273
Performance status: 0 vs. 1	0.846	1.05	0.84–1.31	0.684	Begg's test = 0.734; Egger's test = 0.379
Stage: III vs. IV	0.367	0.88	0.63–1.23	0.443	Begg's test = 0.734; Egger's test = 0.382
Residual disease after surgery: NO vs. YES	0.292	0.85	0.65–1.11	0.219	Begg's test = 0.734; Egger's test = 0.535
Primary vs. interval surgery	0.685	0.92	0.71–1.18	0.501	Begg's test = 1.000; Egger's test = 0.944
**Platinum-free interval**					
>12 vs. 6–12 months	0.909	1.08	0.79–1.48	0.629	Begg's test = 1.000; Egger's test = 0.705
>12 vs. >6 months	0.851	1.03	0.78–1.35	0.843	Begg's test = 0.296; Egger's test = 0.397
6–12 vs. >6 months	0.804	0.98	0.73–1.32	0.911	Begg's test = 0.296; Egger's test = 0.365
Platinum-free interval: >12 vs. 6–12 months	0.909	1.08	0.79–1.48	0.629	Begg's test = 1.000; Egger's test = 0.705
Previous bevacizumab use: YES vs. NO	0.538	1.22	0.76–1.97	0.409	Begg's test = 1.000; Egger's test = /
Race: white vs. non-white	0.839	1.16	0.81–1.66	0.417	Begg's test = 1.000; Egger's test = 0.524

*HR, Hazard Ratio; CI, Confidence Interval; PFS, Progression-Free Survival; CR, Complete response; PR, Partial response; HRD, Homologous Recombination Deficiency. “/”, Not applicable due to limited number of studies*.

In-depth analysis was performed to explore the effect of time to disease progression after a previous platinum therapy (namely PFI) on the efficacy of PARP inhibitor. The results indicated that PARP inhibitors significantly improved PFS for ovarian cancer with PFI of >12 months, 6–12 months, and >6 months (HR = 0.39, 95% CI = 0.31–0.48; HR = 0.40, 95% CI = 0.27–0.57; HR = 0.38; 95% CI = 0.32–0.44, respectively). Interaction tests showed that there were no differences in PFS for PARP inhibitors of >12, 6–12, and >6 months (all *P*_interaction_ for paired comparisons >0.50, Table 3). Pooled within-trial interaction HRs also confirmed the interaction results (>12 vs. 6–12 months: HR = 1.08, 95% CI = 0.79–1.48; >12 vs. >6 months: HR = 1.03, 95% CI = 0.78–1.35; 6–12 vs. >6 months: HR = 0.98, 95% CI = 0.73–1.32, Table 3). Thus, there was no significant differences in PFS for PARP inhibitors between different cut-off values (6 vs. 12 months) for a clinical definition of platinum-sensitive ovarian cancer.

However, for the studies including platinum-resistant patients or mixed platinum-sensitive and platinum-resistant patients, PARP inhibitors did not improve PFS in ovarian cancer (HR = 0.93; 95% CI = 0.76–1.13). The pooled within-trial interaction HR was 1.89 (95% CI = 1.19–3.03) in the platinum-resistant group when compared to the platinum-sensitive group.

### OS for PARP Inhibitors

**Figure 4:**
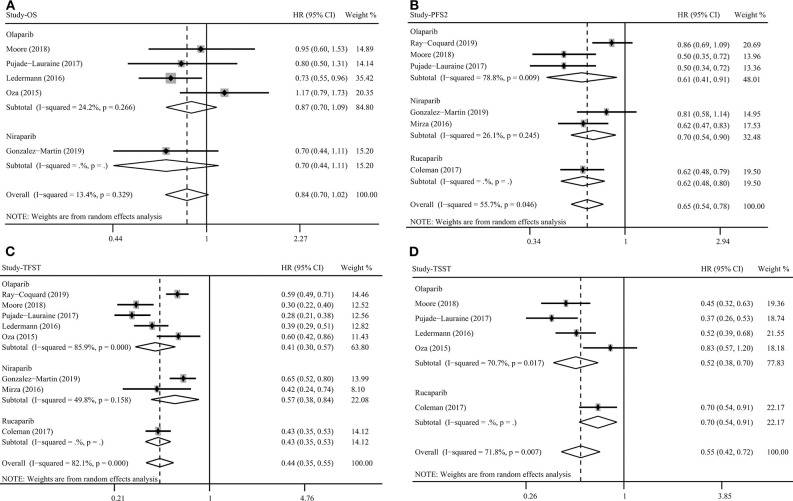
Overall survival **(A)**, progression-free survival 2 **(B)**, time to first subsequent therapy **(C)**, and time to second subsequent therapy **(D)** benefit from PARP inhibitors in patients with ovarian cancer.

A total of five studies assessed the outcome of OS for PARP inhibitors. Pooled results indicated that PARP inhibitors did not significantly prolong OS in patients with ovarian cancer (HR = 0.84, 95% CI = 0.70–1.02, Figure 4). Subgroup analysis for olaparib (HR = 0.87, 95% CI = 0.70–1.09) and BRCA mutation (HR = 0.78, 95% CI = 0.61–1.01) obtained similar results (Table 2). Although the OS benefit from PARP inhibitors was limited, the results should be interpreted with caution, considering that they had a favorable trend and were only marginally insignificant at a 95% CI level. Moreover, the pooled within-trial interaction HR also showed that the BRCA mutation status could not be used as a prognostic factor for OS (HR = 0.74, 95% CI = 0.46–1.18). Sensitivity analysis showed that the clinical trial by Oza et al. (40) slightly affect the overall pooled result (Figure 3). A possible reason for this result is that the Oza et al. (40) trial used PARP inhibitors in a treatment setting, which was different from other four clinical trials. Interestingly, after excluding the clinical trial by Oza et al. (40), the remaining four clinical trials used PARP inhibitors as monotherapy in a maintenance therapy setting after first or subsequent line therapy and the pooled result indicated that PARP inhibitors used as maintenance therapy after first or subsequent line therapy improved OS (HR = 0.77, 95% CI = 0.63–0.93).

### PFS2, TFST, TSST, and CFI for PARP Inhibitors

PARP inhibitors significantly increased PFS2 (HR = 0.65, 95% CI = 0.54–0.78, Figure 4), TFST (HR = 0.44, 95% CI = 0.35–0.55, Figure 4), TSST (HR = 0.55, 95% CI = 0.42–0.72, Figure 4), and CFI (HR = 0.43, 95% CI = 0.35–0.53) in ovarian cancer regardless of BRCA and HRD status (Table 2). Subgroup analysis based on PARP inhibitor type, treatment type, and treatment regimen also showed a favorable PFS2, TFST, and TSST in patients treated with PARP inhibitors (Table 2). Most of the included studies on these outcomes performed an evaluation of PARP inhibitors in a maintenance therapy setting. The present study evaluated the impact of therapy line on efficacy in a maintenance therapy setting and the results showed that PFS2, TFST, and TSST were significantly longer in the PARP inhibitor group than in the control group when PARP inhibitors were used for maintenance therapy after first-line and ≥2-line chemotherapy.

Significant BRCA-related differences in TFST and TSST for PARP inhibitors were observed between BRCA mutation and BRCA-wild groups (TFST: *P*_interaction_ = 0.011, pooled within-trial interaction HR = 0.62, 95% CI = 0.43–0.91; TSST: *P*_interaction_ = 0.012). However, related analysis of PFS2, HRD status, and other clinicopathological factors could not be performed due to the lack of eligible studies.

## Discussion

It is commonly recognized that ovarian cancer is characterized by a remarkable degree of genomic disarray with a lot of mutations (17). Indeed, based on the synthetic lethality of PARP in HRD cancer cells, PARP inhibitors have exhibited favorable therapeutic efficacy for patients with ovarian cancer in several clinical trials (14–16). In clinical practice, PAPR inhibitors have changed the landscape of ovarian cancer treatment. However, prognostic factors for efficacy of PARP inhibitors in ovarian cancer remain unknown. To the best of our knowledge, this is the first meta-analysis to explore the prognostic factors for efficacy of PARP inhibitors in ovarian cancer and to evaluate comprehensive efficacy outcomes, including PFS, OS, PFS2, TFST, TSST, and CFI.

A total of 20 prospective studies containing a total of 6,133 patients with ovarian cancer were included. PARP inhibitors significantly prolonged PFS in patients with ovarian cancer, regardless of BRCA and HRD status. Subgroup analysis based on PARP inhibitors, therapy type, treatment regimens, and clinicopathological factors also showed a significantly improved PFS. Moreover, BRCA mutation, HRD-positive status, and sensitivity to platinum represented important prognostic factors for PFS. However, there was no significant difference between BRCA mutations and HRD-positive status and between BRCA1 and BRCA2 mutations. Other clinicopathological factors, including response to platinum-based chemotherapy (CR vs. PR), PFI, surgery type, residual disease status after surgery, tumor stage, patient age, performance-status score, and race, could not predict the PFS benefit from PARP inhibitors. In addition, PARP inhibitors significantly increased PFS2, TFST, TSST, and CFI in ovarian cancer and there were significant BRCA-related differences. Nevertheless, PARP inhibitors did not significantly prolong the OS in patients with ovarian cancer, although the results had an obviously favorable benefit with a marginal statistical insignificance at 95% CI (HR = 0.84, 95% CI = 0.70–1.02).

Useful prognostic factors were critical for guiding the use of PARP inhibitors in clinical practice, but few effective prognostic factors have been identified until now. Although BRCA mutation is the first and widely-used genotypic prognostic factor for efficacy of PARP inhibitors in ovarian cancer, it was not enough to predict the efficacy of PARP inhibitors. Thus, additional prognostic factors are urgently required. The present results found that both HRD-positive status and sensitivity to platinum represented important prognostic factors for PARP inhibitors. On the basis of the mechanism of synthetic lethality, researchers have realized that HRD is more widespread in ovarian cancer than BRCA. This is because HRD is caused not only by deleterious BRCA mutations, but also by genomic alterations and/or epigenetic inactivation of the BRCA gene and other deficiencies independent of BRCA (known as “BRCAness”) (18, 45) and is associated with efficacy of PARP inhibitors (17, 46, 47). The ENGOT-OV16/NOVA trial found that the PFS benefit of niraparib used as a maintenance therapy in HRD-positive patients was greater than that in HRD-negative patients even in HRD-positive patients without the BRCA mutation (38). The PAOLA-1 trial showed a greater PFS benefit from olaparib in both HRD-positive and HRD-positive with BRCA-wild groups when compared to the HRD-negative group (19). For platinum sensitivity, integrated data analysis from Study 10 and ARIEL2 showed that sensitivity to platinum was significantly associated with favorable PFS in BRCA-mutant ovarian cancer patients treated with rucaparib (35). Understandably, platinum caused tumor cell death by inducing double-stranded breaks. Thus, HRD was more widespread in platinum-sensitive than in platinum-resistant ovarian cancers (17, 18, 48). Indeed, the present pooled results also confirmed the above results. HRD and sensitivity to platinum may be favorable indicators for extending PARP inhibitors to non-BRCA-mutated ovarian cancer. In clinical practice, whether routine tumor clinicopathological factors could play an important role in the PARP inhibitor therapy strategy remains unclear. Furthermore, there are no studies that systematically evaluate clinical values of clinicopathological factors in the PARP inhibitor treatment. Thus, the present study was performed to provide a comprehensive overview of eligible clinical trials, an overall summary of their findings, and a greater understanding of their association strength. Unfortunately, the present results indicated that other clinicopathological factors did not effectively predict the efficacy of PARP inhibitors. A possible reason for this was that PARP inhibitors translated into an extremely favorable PFS benefit with a 56% lower risk of disease progression and thus the role of clinicopathological factors in efficacy of PARP inhibitors may be difficult to detect. Another explanation may be that PARP inhibitors led to cell death by synthetic lethality on a genetic level, weakening the role of clinicopathological factors. The present study focused on one of the main clinical needs in ovarian cancer treatments and thus these results can provide significant information for the clinical application of PARP inhibitors despite negative results. Further studies are urgently needed to explore the effective prognostic factors for the efficacy of PARP inhibitors, which will help PARP inhibitors to be used in a better-suited group of ovarian cancer patients and potentially generate more optimal therapeutic strategies.

The clinical cut-off value for the definition of platinum-sensitive ovarian cancer is relapse 6 months after platinum-based chemotherapy, which is one of the current criteria for PAPR inhibitor prescription for ovarian cancer. However, a population with PFI > 6 months actually refers to a wide heterogeneous group that includes platinum-sensitive (PFI > 12 months) and partially platinum-sensitive (PFI = 6–12 months) patients (49). This categorization may be questionable for the use of PAPR inhibitors in clinical practice. Thus, subgroup analysis and interaction tests were performed and within-trial interaction HRs were determined in order to evaluate the effect of the cut-off value on PAPR inhibitor efficacy. Present results consistently showed no differences in PFS for PARP inhibitors >12, 6–12, and >6 months. Thus, there was no difference between the cut-off values of 6 and 12 months for the PAPR inhibitor prescription. Moreover, further large-scale, prospective clinical trials with homogeneous patients are certainly needed to explore which categorization of the previous chemotherapy benefit is more helpful for PARP inhibitor treatment.

OS remains one of the most important clinical outcomes to evaluate the efficacy of anti-tumor drugs in clinical trials because OS is an unambiguous and unbiased end point and positive results can provide confirmatory evidence that a given drug prolongs the life of a patient (50). However, the effect of PARP inhibitors on OS improvement in ovarian cancer was still inconclusive. The trial in Study 19 reported that olaparib maintenance monotherapy had a longer OS (HR = 0.73, 95% CI = 0.55–0.96), supporting the reported PFS benefit (39). However, SOLO1 and SOLO2 trials showed that the use of maintenance therapy with olaparib did not translate improved PFS into a significant OS benefit among patients with newly diagnosed BRCA-mutant ovarian cancer and platinum-sensitive BRCA-mutant relapsed ovarian cancer patients (14, 15). Besides, the PRIMA trial also provided a significant PFS improvement without significant OS improvement among newly diagnosed ovarian cancer patients treated with niraparib maintenance therapy (20). Indeed, the present pooled results indicated that PARP inhibitors did not significantly prolong OS in patients with ovarian cancer, although a marginally favorable OS benefit was present. A possible explanation for the difference between PFS and OS was the effect of crossover and post-progression therapies (2). In clinical practice, patients may not need to receive immediate subsequent treatment due to disease progression because clinical decision-making should integrate comprehensive clinical information regarding patient's physical condition, tumor condition, and clinical symptoms. The time to subsequent treatment may be more clinically important than the time to progression for patients. The Study 19 trial showed that median PFS was 8.4 and 4.8 months and median TFST was 13.4 and 6.7 months for olaparib and placebo groups, respectively (13, 39). SOLO1, SOLO2, and PRIMA trials obtained similar results for the differences between median PFS and TFST (14, 15, 20). The present results indicated that PARP inhibitors can extend PFS2, TFST, and TSST. Most clinical trials did not provide a mature result for OS and thus the result may be slightly affected by a single trial. Therefore, further studies are needed to determine how to prolong the follow-up duration and to assess the OS benefit from PARP inhibitors using mature OS data.

PARP inhibitor-based treatment strategies for the treatment or maintenance settings and combination treatment or monotherapy are research hotspots in the field of ovarian cancer treatment. Published clinical trials and the present pooled results have consistently found that PARP inhibitors significantly improve PFS in both treatment and maintenance settings. Interestingly, regardless of the number of previous lines of treatment, the favorable efficacy of PARP inhibitors for treatment and maintenance remains strong, indicating that PARP inhibitors can be used during different treatment stages with good safety profiles. Pre-clinical studies have demonstrated that several signaling pathways (i.e., angiogenesis, RAS, PI3K, mTOR, and androgen receptor signaling pathways) participated in the repair of homologous recombination. Thus, drugs targeting these signaling pathways had a potential to chemically induce the HRD phenotype and then acted in synergy with PARP inhibitors leading to synthetic lethality (51). The NSGO-AVANOVA2 trial compared niraparib and bevacizumab vs. niraparib alone in platinum-sensitive recurrent ovarian cancer. The results demonstrated that niraparib and bevacizumab significantly improved PFS compared to niraparib alone, while BRCA-wild patients experienced a greater relative benefit than BRCA-mutant patients (32). A clinical trial by Liu et al. also found that a combination of cediranib and olaparib significantly extended PFS compared to olaparib alone in relapsed platinum-sensitive ovarian cancer, particularly in a BRCA-wild subpopulation (42, 52). However, when and how to best use PARP inhibitors for patient survival remains unclear. Therefore, further studies are needed to explore the best treatment strategy based on PARP inhibitors, especially how to combine them with antitumor drug.

There were several limitations in the present study. First, as a retrospective study, confounding factors and relevant bias could not be controlled or avoided because detailed individual data could not be obtained and the study was performed based on the published data. Second, heterogeneity was present among the studies. Although a relative conservative random-effects model was used to pool the clinical trials, heterogeneity could not be definitively eliminated and explained even in the subgroup analysis. This ineffaceable heterogeneity may result from differences in tumors and host characteristics and other confounding factors. Third, the number of included studies was limited, which affected the statistical power of the subgroup analysis results. In addition, an in-depth subgroup analysis for exploration of prognostic factors could not be performed.

In conclusion, PARP inhibitors can significantly prolong PFS, PFS2, TFST, TSST, and CFI in ovarian cancer patients. BRCA mutation, HRD-positive status, and sensitivity to platinum can predict the efficacy of PARP inhibitors in ovarian cancer. However, there was no significant difference between BRCA mutations and HRD-positive status and between BRCA1 and BRCA2 mutations. Other clinicopathological factors, including response to platinum-based chemotherapy (CR vs. PR), PFI, surgery type, residual disease status after surgery, tumor stage, patient age, performance-status score, and race, cannot significantly predict the PFS benefit resulting from PARP inhibitors. In addition, PARP inhibitors used as a maintenance therapy after first or subsequent line therapy improved OS. The present study focused on one of the main clinical needs in ovarian cancer treatments and provides significant information for clinical application of PARP inhibitors. These results can provide a direction for future research to explore effective and precise prognostic factors for PARP inhibitor efficacy. Thus, further multicenter, large-scale, prospective clinical studies are urgently required to explore effective and precise prognostic factors for the efficacy of PARP inhibitors that can help with individualized PARP inhibitor treatment and to extend PARP inhibitor use to a better-suited population among ovarian cancer patients. In addition, optimal combination treatment strategies based on PARP inhibitors for treatment and maintenance settings are urgently needed in future studies.

## Data Availability Statement

All datasets presented in this study are included in the article/ supplementary material.

## Author Contributions

XH, HJ, and XZ were responsible for conception and design of the study. All authors contributed to the acquisition, analysis, interpretation of data, manuscript drafting and revising, final approval of the version to be submitted and published and agreement to be accountable for all aspects of the work in ensuring that questions related to the accuracy or integrity of any part of the work are appropriately investigated and resolved.

## Conflict of Interest

The authors declare that the research was conducted in the absence of any commercial or financial relationships that could be construed as a potential conflict of interest.
